# Keratinocyte Skin Cancers in General Surgery: The Impact of Anaesthesia, Trainee Supervision, and Choice of Reconstruction

**DOI:** 10.1155/2021/5537273

**Published:** 2021-04-13

**Authors:** William McSweeney, Matthew Leaning, Darius Dastouri

**Affiliations:** Caboolture Hospital, 120 McKean Street, Caboolture 4310, QLD, Australia

## Abstract

**Background:**

Keratinocyte skin cancers are common in Australia, incurring disproportionately high health expenditure in comparison with mortality. General surgeons often excise these lesions as day-surgery. Balancing individual complexities of these cancers with trainee supervision and health expenditure is key to deliver efficacious care and maintain day-surgery volume for patients during a pandemic.

**Methods:**

A retrospective, cross-sectional study was performed, examining 414 procedures from January 2019 to December 2020. Pathology was reviewed, and benign lesions excluded. Complete excision was based on 5 mm margins for squamous cell carcinoma (SCC), 0.5 mm microscopic margins for low-risk basal cell carcinoma (BCC) subtypes, and 3 mm for high-risk. Results of trainee-performed local anesthetic (LA) excision and general anesthetic (GA) excision (consultant scrubbed) were compared.

**Results:**

288 excisions were reviewed for completeness, location, and reconstruction modality. 69% were BCC (199), and 31% were SCC (89). These were excised under GA (72.5%) and LA (27.5%). 25.6% of BCC excisions were “close,” and 22.6% were “positive” under GA, whilst 31% were “close” and 15.5% were “positive” under LA. 52.8% of SCC excisions were “close,” and 7.8% were “positive” under GA, compared with 42.8% “close” and 9.5% “positive” under LA. Complex reconstruction (skin graft, flap) was more common under GA (38% SCC and 36.1% BCC), but occurred at a modest rate under LA (22% BCC and 28.5% SCC).

**Conclusions:**

The results confirm that comparable margins and reconstruction options are achievable when excising keratinocyte cancers under LA by surgical trainees. This is fundamental in cost and timesaving, as well as reducing risk of aerosolisation of virus during GA, in a pandemic.

## 1. Introduction

Keratinocyte skin cancers, which encompass basal cell carcinoma (BCC) and squamous cell carcinoma (SCC), are extremely common in Australia. They also incur a disproportionate cost to the health system, despite their low mortality [1]. This probably stems from the considerable morbidity of these cancers because despite a significant proportion being managed in primary care, they incur the most Australian hospital admissions of any cancer [1]. These cancers are more common in males, in advanced age, and in certain geographical areas such as Queensland [2]. With the demand for excision of such a magnitude, general surgeons are well placed to contribute to the excision of keratinocyte cancers in the inpatient setting.

The primary modality of treatment for surgically resectable keratinocyte cancers is surgical excision, with most being managed in this manner. This has the advantage of a high rate of cure and local control, as well as histological confirmation of the diagnosis. Histologic subtype is paramount when interpreting pathology of surgical excisions and generally divides BCC into “normal” and “high” risk subtypes, based on the Australian Cancer Council [3]. Those considered to be high-risk include infiltrative, sclerosing, micronodular, and basosquamous subtypes, as well as the presence of perineural invasion and recurrent lesions [3]. An adequate microscopic margin for “normal” risk BCC is suggested to be 0.5 mm, whilst a margin of greater than 3 mm is recommended for “high” risk BCC. SCC is typically considered completely excised with a 5 mm margin, based on the Australian Cancer Council.

With the majority of these excisions being done under local anaesthesia, inpatient departments can make meaningful strides in maintaining high-volume throughput, saving operating time and cost, whilst achieving adequate local control regardless of reconstructive method. Whilst this only applies to smaller and less-complex lesions, this represents the vast majority of keratinocyte cancers that are encountered. During the current coronavirus disease 2019 (COVID-19) pandemic, regional anaesthesia incurs not only the multitude of benefits to patient safety but also improved operating theatre efficiency, reduced airway handling, and reduced potential for contamination [3]. The aim of this study was to identify challenges to achieving adequate margins in keratinocyte cancer excisions and whether surgical trainees can reliably achieve these with local anaesthesia. During times of significant strain on service delivery, the majority of “minor operations” lists would appear to be a logical first-choice in scaling-back provisions, but this may not be the simple answer it would appear to be. The secondary aim of this review was to propose an alternative rationale of increased utilization of “minor operations” lists, or LA-predominant lists, which save health service expenditure and allow for development of these basic surgical competencies for trainees.

## 2. Methods

A retrospective, cross-sectional, observational study was performed at a single institution within the general surgical department. We examined 414 procedures from January 2019 to December 2020. Pathology was reviewed, and benign lesions were excluded. Complete excision was based on 5 mm margins for squamous cell carcinoma (SCC), 0.5 mm microscopic margins for “normal” risk basal cell carcinoma (BCC) subtypes, and 3 mm for “high” risk. Results of trainee-performed local anesthetic (LA) excision (performed by trainee with consultant supervision) and general anesthetic (GA) excision (consultant scrubbed and operating) were compared. The location of the lesion was recorded, as well as the modality of reconstruction (skin graft, flap repair, or primary closure). Results of excision margins were grouped by histology and then denoted as complete, close margin, or positive margin. Only BCC with close margin (less than 3 mm on initial review) were reviewed further to identify histological subtype and then denoted as either “normal” or “high” risk, to determine whether the margins were in fact adequate.

## 3. Results

288 excisions were reviewed individually with pathology for complete excision, location, and reconstruction modality. The excisions were carried out under local anaesthesia in 27.5% (*n* = 79) and general anaesthesia in 72.5% (*n* = 209). Histology of excision specimens revealed 69% were BCC (*n* = 199) and 31% were SCC (*n* = 89). 25.6% of BCC excisions were “close” and 22.6% “positive” when excised under GA, whilst 31% were “close” and 15.5% were “positive” when excised under LA. Of those with close margins, the vast majority were on the head and neck (*n* = 38, 74.5%), followed by lower limb (*n* = 8, 15.6%) and finally the upper limb and torso (*n* = 1, 1.9% and *n* = 4, 7.8%). Those with a positive margin followed a similar trend, with the vast majority on the head and neck (*n* = 40, 88.9%). Reconstructive modalities employed for BCC were primary closure (*n* = 127, 63.8%), skin graft (*n* = 40, 20.1%), and flap repair (*n* = 32, 16.1%).

52.8% of SCC excisions were “close” and 7.8% “positive” under GA, compared with 42.8% “close” and 9.5% “positive” under LA. Similar to BCC, the majority of SCC with close margin was on the head and neck (*n* = 25, 53.2%), followed by the lower limb (*n* = 16, 34%), upper limb (*n* = 5, 10.6%), and torso (*n* = 1, 2.1%). Positive margins were again much more commonly encountered on the head and neck (*n* = 6, 85.7%). Reconstructive modalities were similar, with primary closure most common (*n* = 55, 61.8%), followed by skin graft (*n* = 25, 28.1%) and flap repair (*n* = 9, 10.1%).

Complex reconstruction (skin graft, flap) was more common under GA (38% SCC and 36.1% BCC), but occurred at a modest rate under LA (22% BCC and 28.5% SCC).

## 4. Discussion

This study reports on the achieved surgical margins of excision of keratinocyte cancers across a wide range of locations. The rate of positive margin or incomplete excision was higher than recent publications of both SCC and BCC [4–6]. This is multifactorial, as a number of lesions in this review were not biopsied preoperatively. Additionally, lesions across all regions were included. Preoperative identification of high-risk subtype BCC would potentially substantially reduce the rate of incomplete excision by more accurate preoperative marking and operative planning. In addition, head and neck regions are often challenging to achieve high rates of complete excision with adequate margins [4].

During the COVID-19 pandemic, surgical resource allocation and planning is at the forefront of service delivery. It is essential that services attempt to minimize risks associated with general anaesthesia to patient and importantly, to staff. These risks include aerosolisation during airway manipulation, as well as increased exposure to patient secretions. General anaesthesia also necessitates a more frequent consumption of personal protective equipment (PPE) and prolongs utilization of operating theatres, which may already be in demand [7]. This study reports similar rates of close or positive margins for excisions of BCC and SCC under LA, compared with GA. The modalities employed for reconstruction, whilst being predominantly primary closure, were still modestly utilized under LA. This provides more evidence to the notion that general surgical departments, often faced with less-complex lesions than their plastic surgery or dermatology colleagues, can prioritize LA techniques to achieve similar oncological outcomes, cosmetic outcomes, preserve PPE, and reduce risk to staff. This allows for continued delivery of high-volume, efficient care for this highly prevalent disease entity during a pandemic.

Whilst surgical trainees performed local anaesthesia cases in this review predominantly, it is important to note that rates of incomplete or positive margins did not rise sharply. These LA cases are often valued developmental opportunities early in a surgical career and reinforced that trainees are likely vigilant in achieving appropriate margins.

There are numerous limitations of this study. As with other published studies, there may be a degree of bias as more complex resections and reconstructions are more likely to be streamed to a GA operating list and be performed by a consultant [8, 9]. Similarly, lesions of a more complex nature by location or size may be referred by primary care providers preferentially to dermatologists or plastic surgeons. In addition, larger lesions are inherently more likely to be performed on GA operating lists, as this selection is made entirely by discretion of the surgeon in outpatient clinics. Lastly, age and gender were not specifically analysed and accounted for and are certainly contributors to bias as surgeons may opt to perform smaller excisions to preserve cosmesis in younger patients or female patients who anecdotally may be more forthcoming with concerns regarding cosmesis.

Most importantly in this series, LA modality did not dramatically alter the rate of incomplete excision or the choice of reconstruction. Other larger, prospective studies have shown that LA is a feasible choice and that LA as an independent factor did not affect outcome of excision [10].

## 5. Conclusion

This article provides further evidence to guide decision-making regarding resource utilization during challenging economic times. Comparable rates of local control and reconstruction can be achieved with LA techniques to reduce PPE consumption and staff aerosol exposure, as well as maintain high patient turnover and case volume.

## Data Availability

The data used to support the findings of this study are available from the author upon request.
